# Crystal structures of increasingly large molecules: meeting the challenges with CRYSTALS software

**DOI:** 10.1186/s13065-015-0105-4

**Published:** 2015-05-24

**Authors:** Pascal Parois, Richard I Cooper, Amber L Thompson

**Affiliations:** Chemical Crystallography, Chemistry Research Laboratory, Mansfield Road, Oxford, OX1 3TA UK

**Keywords:** Crystals software, Large molecule crystal structures, Least-squares refinement

## Abstract

**Background:**

The size and complexity of molecules being studied by single crystal diffraction is growing year by year, resulting in an increase in the difficulties encountered during structure determination. From the crystallisation itself and sample handling, to structure solution and refinement, specific problems due to larger molecules are discussed.

**Results:**

During refinement, several methods are available to deal with the problems encountered with large structures within the software Crystals. Hydrogens atoms can neither be found easily nor refined freely, but restraints can be applied automatically. Special scattering factors can be used to model complex disorder. Finally chemical information can be included in the form of restraints in order to help the determination of a good model.

Multicollinearity problems are more likely in the refinement of large structures; to some extent more precise and accurate algorithms can help. Also, if the global minimum is less well defined, faster refinement enables more cycles to be carried out, a necessity for good convergence. The efficiency of the algorithms in Crystals have been increased to help address these issues.

**Conclusions:**

Thus, crystal structures are getting larger and their complexity is increasing. Recent developments in precision and speed during the least squares in Crystals is helping the structural scientist to deal with larger structures more efficiently.

## Background

In the century since William Henry and William Lawrence Bragg reported their Nobel prize-winning studies of single crystal X-ray diffraction [1], the determination of chemical structure using the method has become a mature analytical technique. X-ray diffractometers have become increasingly available within university research facilities and data measurement, structure solution and refinement are all now carried out by non-expert users. Whereas fifty years ago it took weeks to collect the data and months to process it, nowadays under the right conditions, an entire dataset can be collected in a matter of minutes and solution and refinement to publication quality can be completed in a similar amount of time. Thus what typically took one year, can now be completed in less than an hour. However, while this is true for the trivial case, there are many examples that are much more complex and scientists continue to push the limits with ever more challenging materials being studied.

**Fig. 1:**
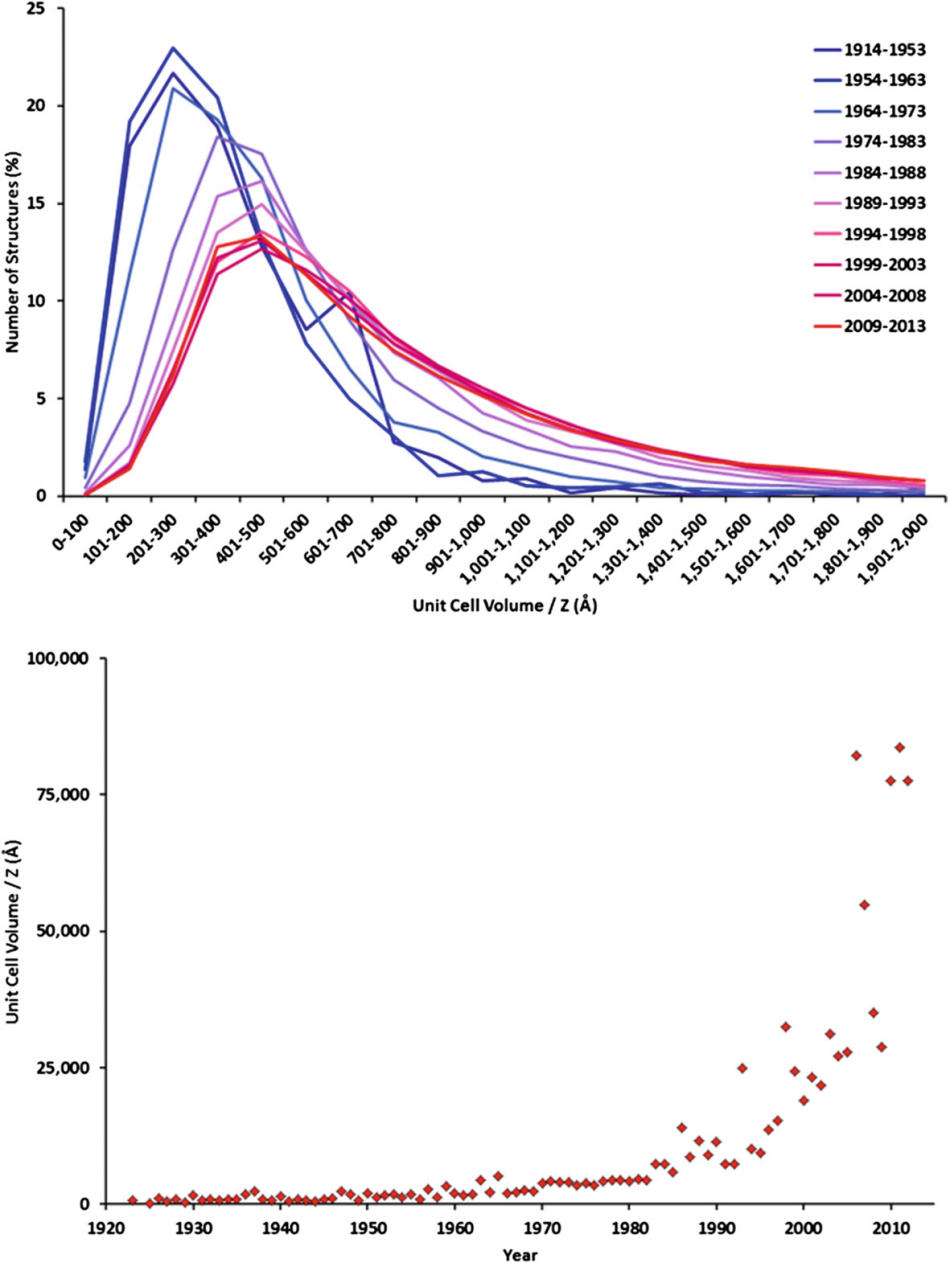
The size of molecules charaterised by single crystal diffraction over the last hundred years. (top) The percentage of structures is shown as a function of size in each time period given. It is clear that the percentage of smaller structures decreases over time (going from blue to red) as the number of larger structures determined increases. (bottom) The size of the largest molecule for each period is shown, showing how the largest molecules reported have also increased over the years

One area where this is more visible than most is the size of molecules studied. Ever larger molecules are being synthesised and characterised and this is reflected in the increased size reported in the Cambridge Structural Database [2, 3] (Fig. 1). Increasing molecular size poses a number of challenges: crystallisation; the experiment itself; and finally solving, refining and validating the structure before publication. These problems will be discussed in detail herein, together with some of the approaches used by the refinement software, CRYSTALS [4], to deal with the advances in this aspect of small molecule crystal structure determination.

## Results and discussion

### The challenges associated with determining the structure of larger molecules

The challenge begins with obtaining viable crystals: large molecules are often more difficult to crystallise. There are several reasons for this. Firstly, larger molecules generally form a more viscous solution and it is harder for them to organise themselves in a regular way. The archetypal “large molecules” are proteins, however, these contain many small but significant interactions leading to the formation of secondary structure domains, which in turn organise into the tertiary structure of the protein. In contrast, synthetic molecules generally do not have such a high density of strong directing interactions. Secondly, the search for appropriate crystallization conditions can be much more extensive. Proteins are typically crystallised from aqueous solutions; the structures of proteins in the absence of water may be of specialist interest [5], but conditions which reasonably closely resemble their *in-vivo* environment are usually desirable. The chemical diversity of synthetic molecules is much larger than for proteins, which means that the ideal solvent for crystallisation of synthetic molecules could come from a list of tens or hundreds, or a mixture thereof, and the search space becomes extensive.

#### Sample handling

Larger molecules often have multiple possible conformations of similar energy. Sometimes these conformations involve a slight alteration in the orientation of small part of the molecule *e.g.* a terminal tertiary butyl group, or a bridging phenyl group. This leads to one of the most time consuming aspects of small molecule crystal structure determination: dealing with disorder [6].

**Fig. 2:**
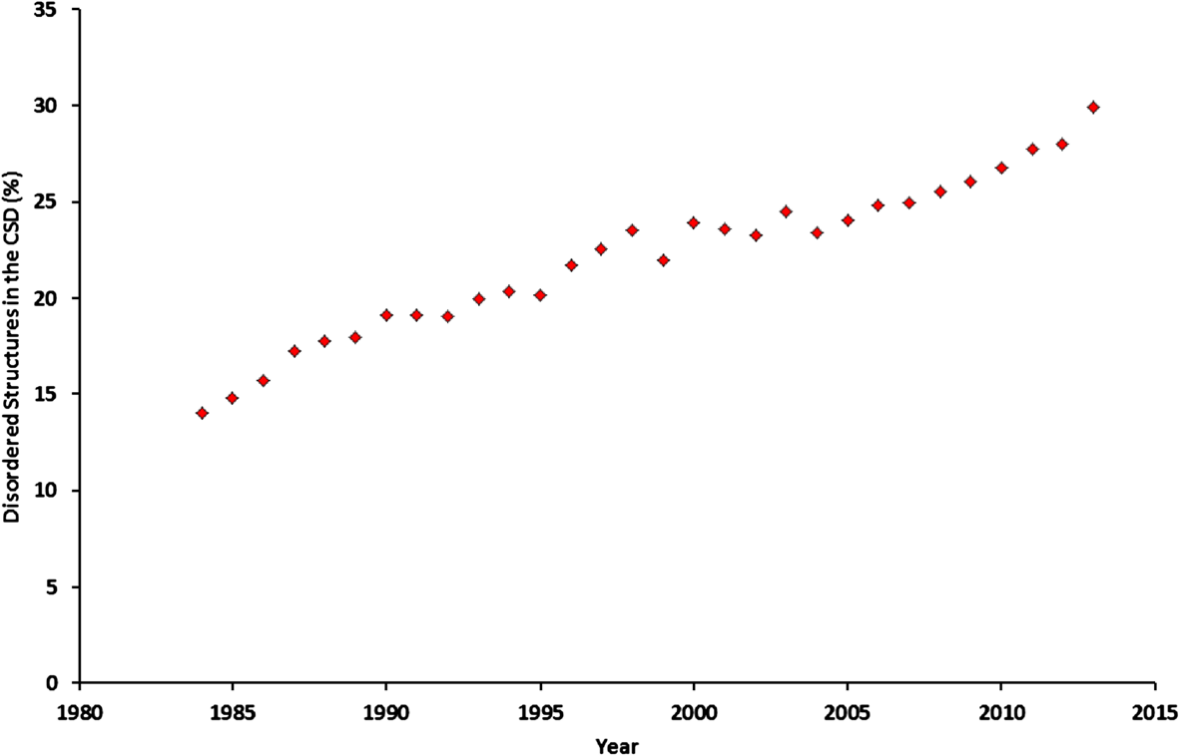
The percentage of structures flagged as disordered in the CSD over the last twenty-five years

Disorder is one of the biggest problems in crystallography as it not only reduces the observable data in a diffraction pattern (due to reduced intensity at high angle) but it also makes fitting electron density more difficult. It is an increasing problem: nearly 30 % of structures published in 2013 and reported in the Cambridge Structural Database [2] were flagged as disordered and the trend over the last thirty years shows that this has more than doubled since 1984 (Fig. 2). While there are some structure analysts who take a perverse pleasure in modelling complex disorder, it is a nightmare for beginners and a pain for others. Since it often does not affect the fundamental chemistry, it is often seen as a nuisance and is certainly a pit to pour time into, which may be why many publications have avoided the subject. However, disorder is sometimes vital to our understanding of the chemistry and, because every parameter in the structural model contributes to the fit to every observed structure factor modulus, it is very important to deal with it properly as incorrect treatment can lead to errors elsewhere in the model.

Trifluoromethyl substituted phenyl groups often exhibit disorder due to the torsional flexibility about the C—C bond which enables the CF _3_ group to rotate, or librate. The difference in energy between the situation where there are two fluorine atoms above the plane of the aromatic ring and one below, and *vice versa*, is negligible. Not only is the energy difference between the two states small, but the barrier to rotation between the states is also small, of the order of *k*_*B*_*T* (where *k*_*B*_ is the Boltzmann constant and *T* is the temperature). Thus, at high temperatures it may possible to move between them, with the result that the CF _3_ group is spinning. This is generally referred to as “dynamic disorder”. At low temperature, there may be no kinetic pathway between the two states giving rise to “static disorder”. This means, that whereas dynamic disorder can be reduced by lowering the temperature of the data collection, generally, changing the measurement conditions will not change the degree of static disorder. Sudden cooling can also quench dynamic disorder to static disorder, as the sudden temperature change traps chemical groups in a local thermodynamic minimum. This has implications for the modern practice of using the oil-drop flash freezing technique to mount samples at low temperature as this could exacerbate problems with disorder.

It may be possible to use thermal cycling to reduce this problem, however this not necessarily advisable as it can introduce other problems – quench cooling may trap a high temperature phase in a metastable form, whereas thermal cycling will allow it to form a low-temperature pseudo-symmetric phase leading to subsequent difficulties in the analysis. Crystals of large molecules can often be difficult to handle and it can be hard work to find diffracting samples at all. Therefore it would be a courageous crystallographer who is prepared to risk subjecting the sample to further environmental stresses once a crystal is on the diffractometer. Nevertheless, there has been success reported using the so-called “credit card” method with protein crystals: a sample is annealed by warming briefly to room temperature by momentarily blocking the cold-stream of the cryo-device using a small piece of plastic or card. Devices have been developed to facilitate this, called “flippers” [7–9].

**Fig. 3:**
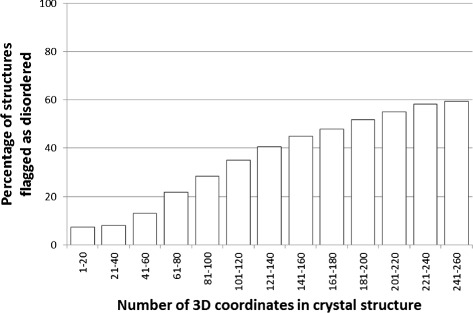
The relationship between the size of structures (reported in the CSD between 2000 and 2009) and the rate of occurrence of disorder

When dealing with crystals of large molecules, solvent loss or sensitivity to air/moisture may necessitate rapid handling and low temperatures, which have additional potential to increase the probability that groups become disordered. With larger molecules the number of possible disordered “assemblies”^1^ increases as shown by a plot of the reported rate of occurrence of disorder amongst crystal structures of different sizes (Fig. 3).

#### Restraints

In CRYSTALS, restraints are stored in LIST 16 and are generally of the form



thus a simple distance restraint might be written



The chance of repeated chemical motifs is much higher in large molecules, which means that rather than restraining a given distance to a specific value, sets of distances and angles can be restrained to be equivalent with a defined tolerance. In CRYSTALS these can be applied very efficiently using the command SAME. In this type of restraint, the first group of atoms named is the “target” and all following groups are mapped onto it in order specified. CRYSTALS then uses the connectivity of the first group to work out which distances and angles should be restrained and effectively decomposes the SAME into a series of DISTANCE and ANGLE restraints. For example the restraints below might be used to restrain two pyridine rings:



In this example, these four lines are decomposed into forty-eight separate DISTANCE and ANGLE restraints which map the geometry of the second ring onto the first and additionally uses the inherent mirror symmetry of the pyridine ring to map the geometry of mirrored versions of the first and second ring back onto the first. Thermal similarity restraints and vibrational restraints (which restrain given displacement parameters to be similar and obey the Hirshfeld test [10]) can similarly be simplified using SIMU and DELU between chemically identical groups.

**Fig. 4:**
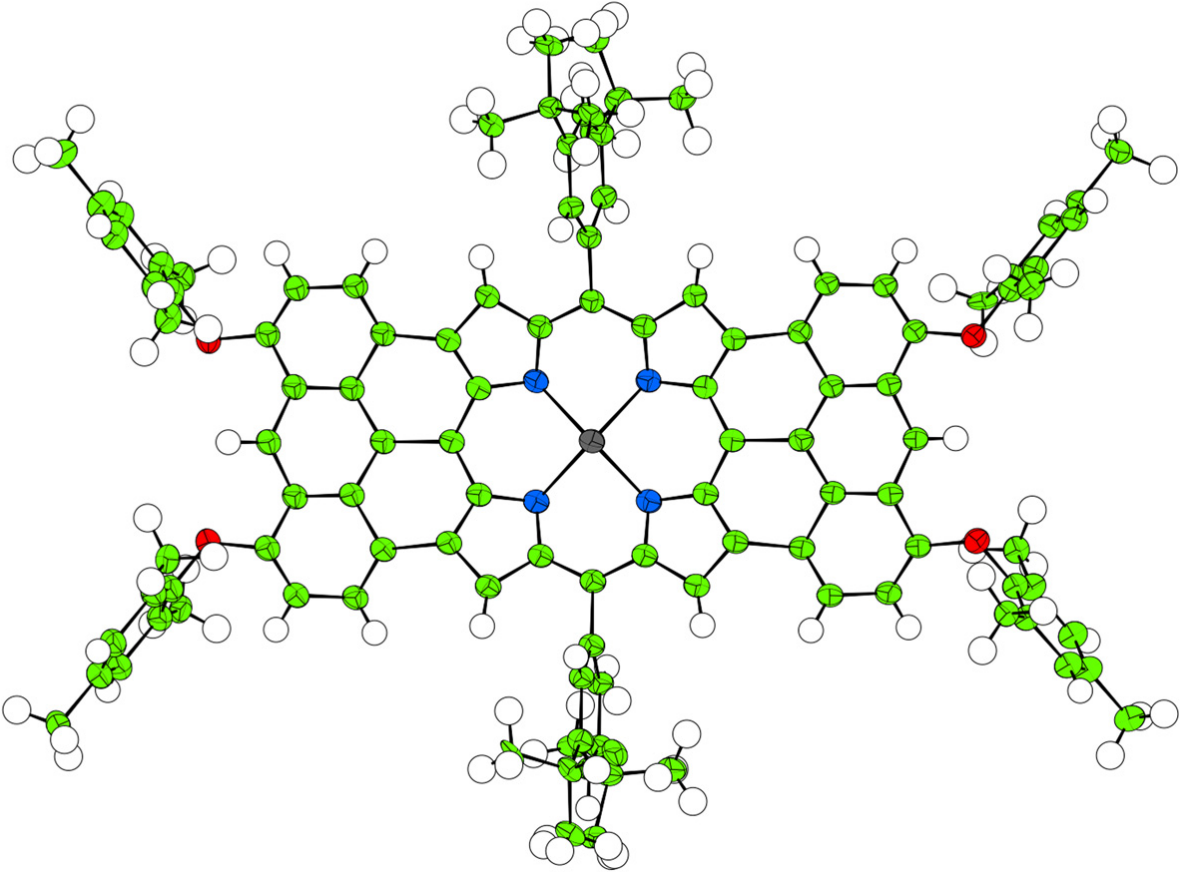
An extended nickel porphyrin structure reported by Davis *et al.* [11]. Displacement ellipsoids drawn at 25 % probability for clarity

In structures where there are persistent repeat motifs, these short-cuts can save an enormous amount of time and increase the accuracy of the model. In the case of the extended nickel porphyrin reported by Davis *et al.* [11] (Fig. 4), good use could also be made of the non-crystallographic approximate *D*_2*h*_ symmetry of the molecule.

**Fig. 5:**
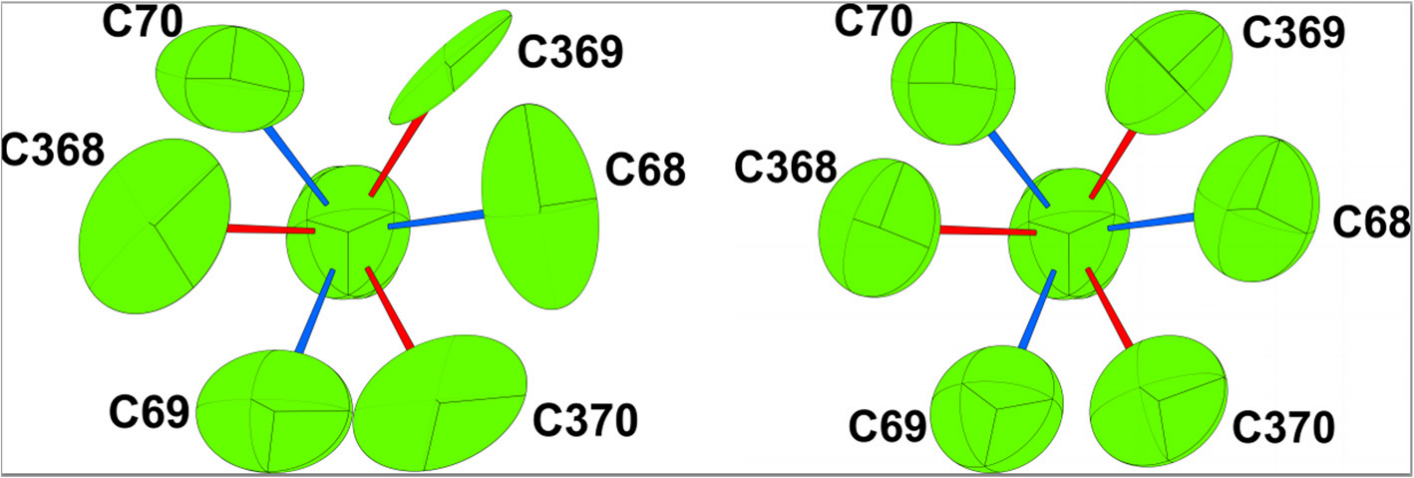
An unrestrained (left) and restrained (right) disordered tertiary butyl group. An unrestrained disordered tertiary butyl group with the hydrogen atoms and rest of the structure omitted for clarity (left). In this case, the two disordered components are shown with red and green bonds and atoms on opposite sides are restrained using asymmetric similarity restraints (right). Thus, C70 is restrained to be similar to C370 etc

The application of such restraints is very powerful, and where component parameters of the restraints are poorly defined they help all components to tend to the average – ultimately giving a better end result. However, in the case where one component is well defined and the other is poorly defined, the average of the two can be a degraded version of the well resolved component. In such cases, the asymmetric restraint [12] as implemented in CRYSTALS, might be a better choice. The geometry or displacement parameters of a putatively unreliable chemical group are restrained to match those of a better defined, identical group in the molecule. Thus, the geometry of the well defined group acts as a reference, but its parameters are not unduly influenced by the poorly defined group which it is being used as a guide. These restraints are very useful when dealing with disordered CF _3_ or tertiary butyl groups where components are disordered and the minor component is significantly less than 50 % occupied. In these cases an asymmetric similar ADP restraint can be applied to atoms in different parts of the disodered fragment related by 180 ° rotation around the C–C bond vector (Fig. 5).

#### Hydrogen treatment

Hydrogen atoms cannot be refined freely in structures of large molecules due to their small X-ray scattering factors, relatively large atomic displacements (resulting from their terminal positions), and overall poor resolution data. However, hydrogen atoms are often visible in the difference Fourier map. In CRYSTALS, hydrogen atoms are added geometrically to sp^1^, sp^2^ and sp^3^ carbon atoms, with the option of adding those bonded to heteroatoms from the difference map [13]. By default CRYSTALS then refines the hydrogen atom positions with soft restraints in a separate least-squares cycle prior to inclusion in the final refinement using a riding model. This approach ensures the best possible fit to the measured data while maintaining a sensible geometry. In the extreme case, even with restraints, refining hydrogen atoms may be unstable, in which case, they can be added geometrically or to satisfy hydrogen bonds (using options within the software).

#### Special shapes

During a refinement, atoms are initially modelled using isotropic displacement parameters and later using a 3×3 tensor describing an anisotropic probability density function (PDF). However, these are often poor approximations of the real displacements of atoms within molecule. For example, the cyclopentadiene ring in a metallocene complex usually librates about an axis described by a vector from the metal to the centre of the ring. Thus the displacement of each individual atom is better described by a curved PDF [14].

In CRYSTALS, three non-standard parameterisations of atom positions are implemented: the spherical shell; the line; and the torus. The spherical shell is the simplest and is described using the position of its centroid, a standard displacement parameter, U _iso_, and an absolute magnitude which corresponds to the radius of the shell. The line and torus require additional descriptors for the orientation and, in the case of the line, the radius parameter is replaced by a length. These are the *declination* (the angle between the line axis or torus normal and the *z* axis of orthogonal coordinate system used in CRYSTALS) and the *azimuth* (the angle between the projection of the line axis or torus normal onto the *x*-*y* plane and the *x* axis of the orthogonal coordinate).

These sets of parameters are typically used to model disordered anions like $\mathrm{PF}_{6}^{-}$ (where the spherical shell is used to model the six fluorine atoms), disordered solvent in channels (line), and most frequently, the torus which can model librating CF _3_ groups, methylcyclopentadiene ligands or benzene. In most cases, a combined model is used where the “special shape” is partially occupied and has conventional partially occupied atoms embedded in it to reflect the fact that the electron density is non-uniform. The high degree of parameter correlation in these combined models means that restraints are required to ensure convergence and to maintain a physically reasonable model.

**Fig. 6:**
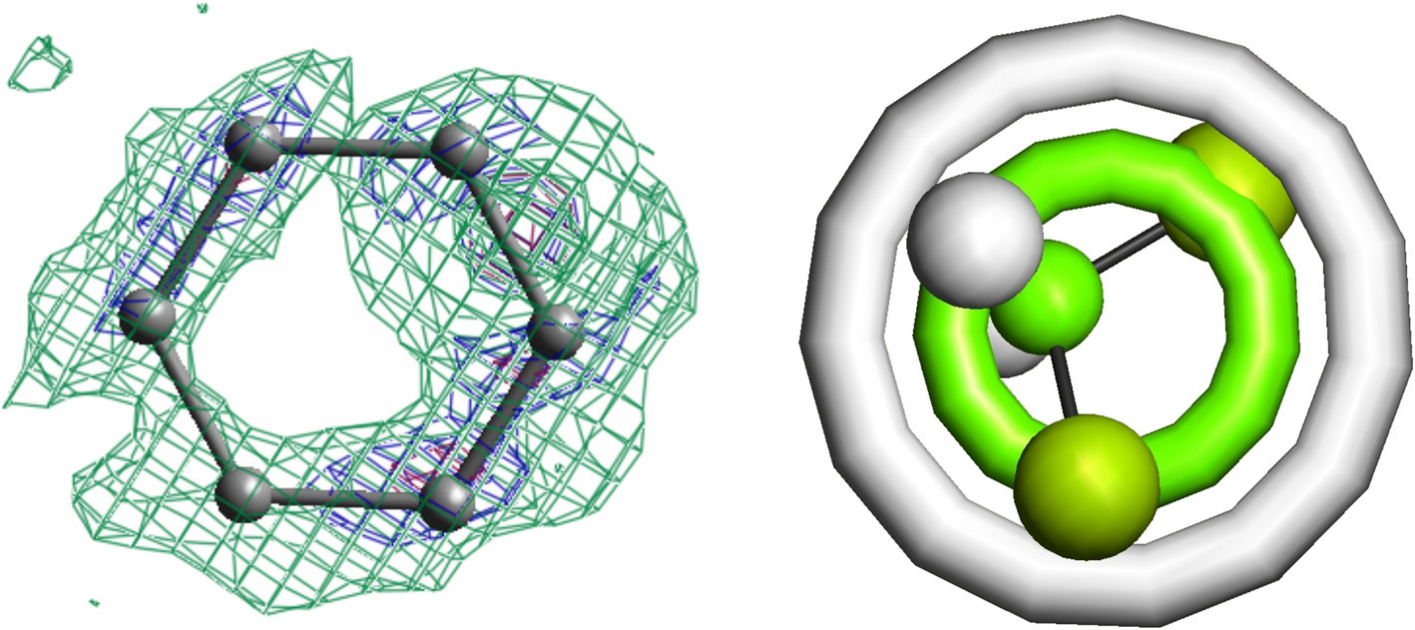
Modelling of disordered electron density using non-atomic scattering factors. Benzene and DCM together showing the electron density (left) and the final model which used a pair of concentric rings with 5 % DCM occupying the same site (right)

This approach was used to great effect modelling a disordered benzene molecule which was included in the crystal structure of another molecule. The principal solvent was identifiable as benzene from the initial structure solution, but was extremely difficult to refine because it exhibits librational disorder (Fig. 6). Initially this was modelled using a two-component disorder model with constraints to maintain physically reasonable positional and displacement parameters. However, a better solution was to use two concentric tori to model the six carbon and six hydrogen atoms. Examination of the difference map for this model showed the presence of two significant peaks just under 3 Å apart. These were modelled as 5 % DCM. Unsurprisingly, free refinement of this model was unstable, so the occupancies and isotropic displacement parameters were fixed to sensible values and the positions, declinations and azimuth parameters of the carbon and hydrogen tori were constrained to be equal.

#### Multicollinearity

Multicollinearity can occur in a least-squares crystallographic model when two or more of the physical parameters are approximately related by a linear function. A simple example would be attempting to refine the relative occupancies of a site containing an unknown mixture of Ga^+^ and Ga^3+^ ions – the X-ray data do not contain enough information (or rather it contains too much noise) to distinguish the two species [15]. As a consequence the relative occupancies of the two ions do not have much influence on the fit of the model to the data, however small changes in the data can cause wild fluctuations in the occupancies of the model, and they can easily take on physically meaningless values, provided that the sum of the two species remains at about unity.

In the case of perfectly multicollinear parameters, or combinations of parameters, the normal matrix **AA**^*t*^ is rank deficient and has no inverse, leading to computational problems carrying out a refinement. Noise in the experimental data makes perfect multicollinearity unlikely in practice and usually an inverse can be computed, though it may be inaccurate and highly sensitive to small errors in the data and small changes in the model.

As the complexity of a crystallographic model increases, the chance of approximate multicollinearity increases: atoms in disordered regions which occupy similar positions in the average structure will have very similar contributions to the structure factor equations; pseudo-symmetry can also result in similar scattering contributions from related parts of the model. At the same time, multicollinearity becomes more of a problem as fewer data are included in the model and this effect can be caused by disorder or solvent or poor crystallinity limiting the X-ray data resolution.

**Fig. 7:**
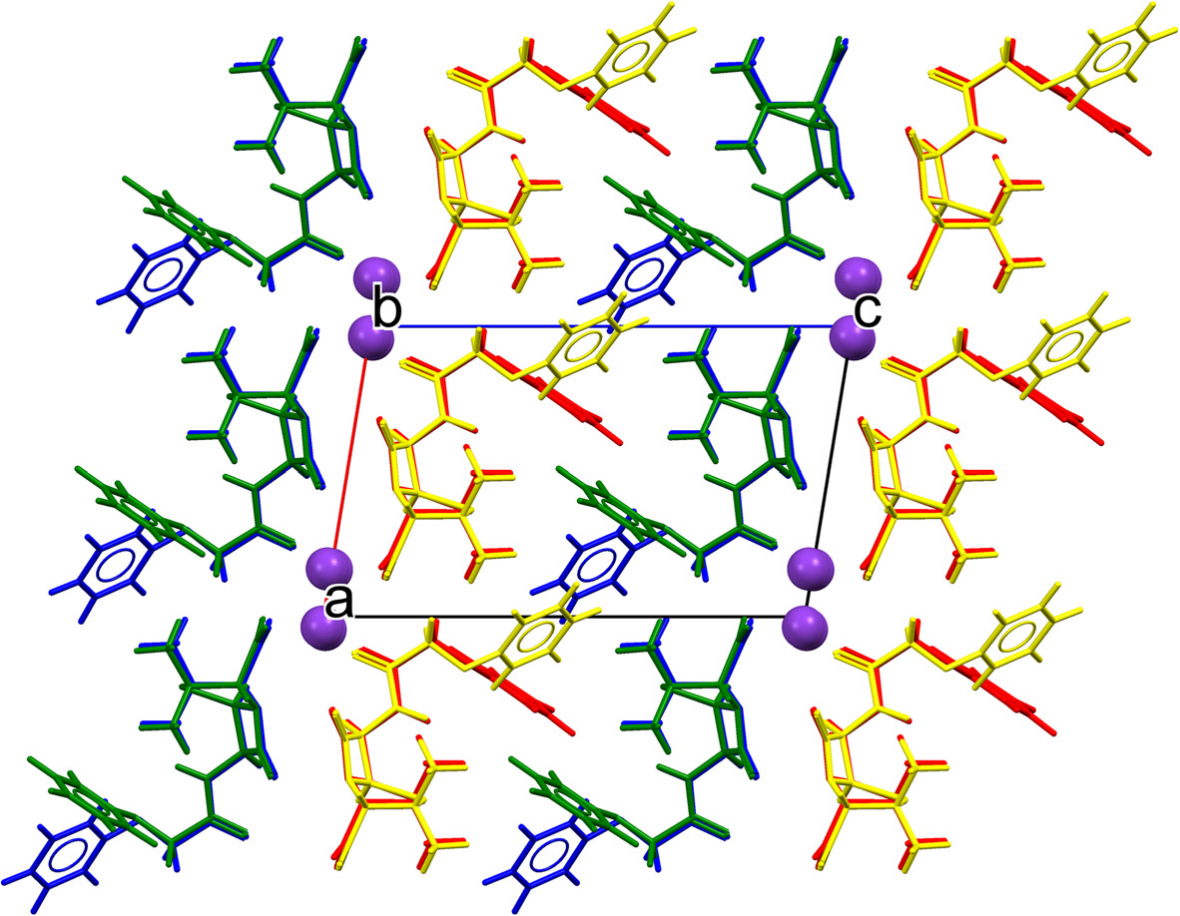
The structure of potassium phenoxymethylpenicillin viewed down the *b*-axis. The asymmetric unit consists of four potassium ions (shown in purple) and four phenoxymethylpenicillin molecules (shown in red, green, blue and yellow). The red and green pair of equivallents of phenoxymethylpenicillin are related by a pseudo 21 screw axis and the red and yellow pair by an approximate translation; the blue and yellow pair and blue and green pair exhibit similar relationships

The presence of pseudo-symmetry [16, 17] is sometimes a problem when trying to refine the structure of a material that undergoes a subtle phase transition – unit cell doubling for example can lead to highly collinear combinations of atom position parameters; the only data about the specific differences between the average and doubled cell are present in the weak superstructure reflections. It has been shown [18] that structures in non-centrosymmetric space groups are more likely to have more than one molecule in the asymmetric unit as these materials mimic the inversion centre with an approximate symmetry operator. For example, the triclinic polymorph of potassium phenoxymethylpenicillin (Fig. 7) [16] exhibits this type of pseudo-inversion symmetry. In addition to the refinement problems associated with pseudo symmetry, these structures are also more challenging simply as a result of size. Potassium phenoxymethylpenicillin becomes a large structure simply because, with four molecules in the asymmetric unit, there are four times as many atoms as in a typical structure of a similar sized molecule.

The increased tendency to disorder and associated resolution reduction in observable X-ray data in structures of large molecules can introduce multicollinearity problems. In such refinements, the minimum may be poorly defined and restraints are added to ensure that the model is chemically sensible. The nature of these chemical data is usually to give very local (atom- and bond-centred) information related to geometry and displacement parameters rather than giving information that defines a global minimum. As a result it can take several cycles of least-squares refinement to find the global minimum with respect to all of the restraints. Consider a single alkyl chain: If all the bonds are 0.1 Å too short, restraining each to the correct length would require several cycles of shifting positions until all the bond length restraints are optimally satisfied. This is because the refinement can only attempt to satisfy the local equation for each bond at every step and the refinement has no way of knowing that the terminal atom will ultimately have to move a total of 0.1 Å for every bond in the chain.

This effect, together with the necessarily poorly defined minimum means the structure will generally be slow to converge, indeed, it may never converge and the atoms will tend to shift by small amounts with each additional cycle of least-squares. The temptation is then to apply more restraints to encourage the refinement to obey preconceived ideas or to apply shift-limiting restraints to bring the refinement to convergence. In fact it is often better to reduce the number of restraints and apply constraints instead, which remove correlated parameters where possible.

**Fig. 8:**
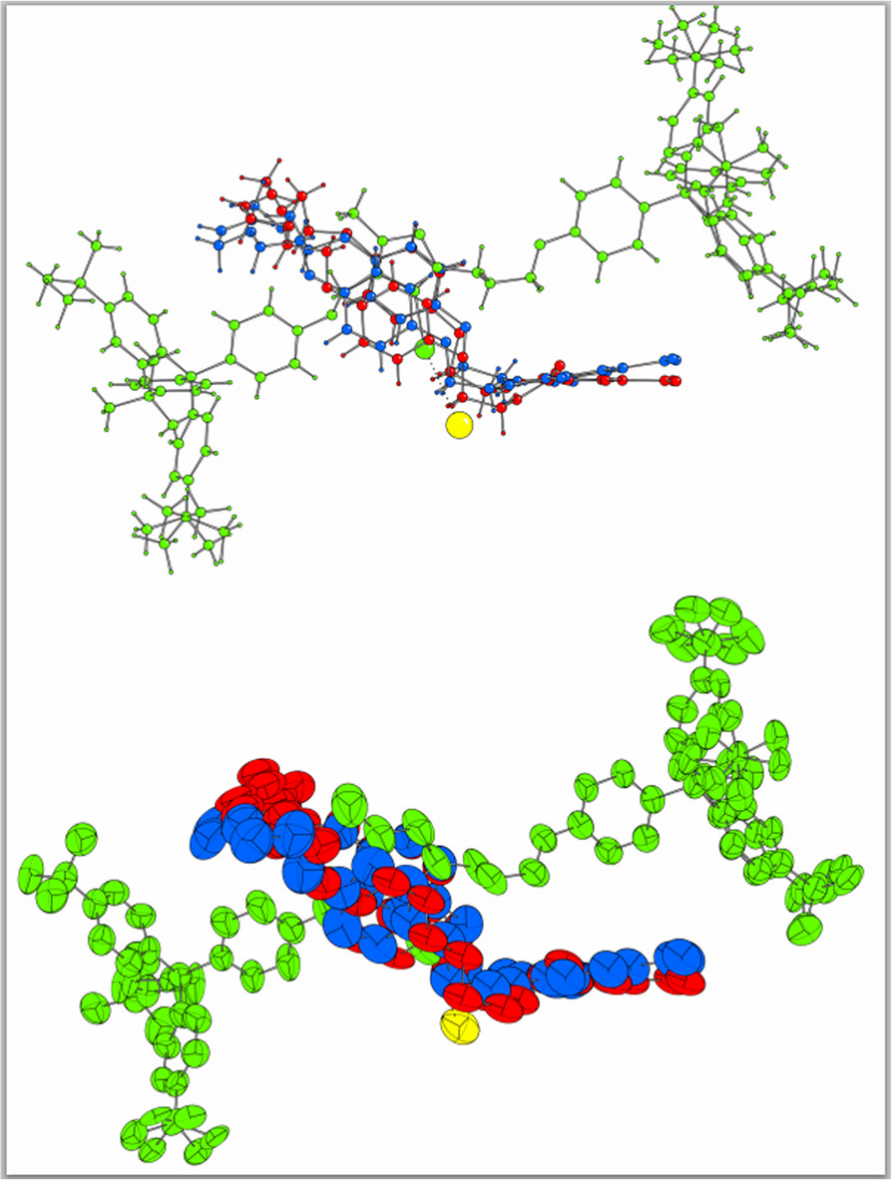
The halogen bonded rotaxane structure. The halogen bonded rotaxane structure is shown (top) with the thread in green, the two disordered components of the macrocyle in red and blue and the I ···Br interaction shown as a dotted line. A displacement ellipsoid plot is shown (bottom) with the hydrogen atoms omitted for clarity. In this structure, constraints were used for atoms in the macrocycle that were in very close proximity to reduce the correlation

One example of a structure exhibiting this type of problem was the first halogen bond templated rotaxane structure [19]. In this material the “stoppers” of the threaded molecule were highly disordered, but the real problem was the macrocycle (Fig. 8). The difference Fourier map and shape of the displacement ellipsoids clearly show that it is disordered, occupying at least two different conformations throughout the crystal structure. The refinement was very slow to converge and was stabilised by careful application of constraints for ADPs that were in very close proximity in the average disordered model. Similarly, constraints were applied to some of the disordered tertiarybutyl groups, constraining ADPs of components related by a 180 ° rotation to be the same as described above (Fig. 5).

#### Solvent

Perhaps one of the biggest problems associated with large structures is the inclusion of disordered solvent molecules within the structure. It is often the case that solvent close to an ordered molecule is reasonably well ordered, but becomes increasingly disordered further away [20]. In extreme cases, the lack of long-range order is so severe that individual atoms cannot be identified. This phenomenon is well known in small-molecule structures, but the problem increases as molecules get larger.

Since invariant and semi-invariant phase relationships of direct methods are derived using the assumption that the electron density is atomic, extreme disorder of solvent may lead to difficulties at the structure solution stage. The advent of charge-flipping methods [21, 22] provides one solution to this problem and SuperFlip [23] has been integrated seamlessly into CRYSTALS [24].

Finding and refining an appropriate model for disordered solvent is still a problem however: it simply cannot be ignored. The minimisation function is

(1) $$ M = \sum (Y_{o} - Y_{c})^{2}  $$

where *Y* denotes either *F* or *F*^2^ and the sum is over all measured *Y*_*o*_. The calculated structure factor magnitude can be separated into the total contributions from the molecule and the solvent as follows:

(2) $$ |F_{c}| = |F_{c_{\text{molecule}}} + F_{c_{\text{solvent}}}|  $$

The solvent contribution must be included in the model to avoid systematic errors in *Y*_*c*_ which will lead to a systematic error in the other parameters because the function minimised during refinement (Equation 1 [25]) contains the observed quantity *Y*_*o*_ which has contributions from all atoms, including solvent, however disordered it may be.

**Fig. 9:**
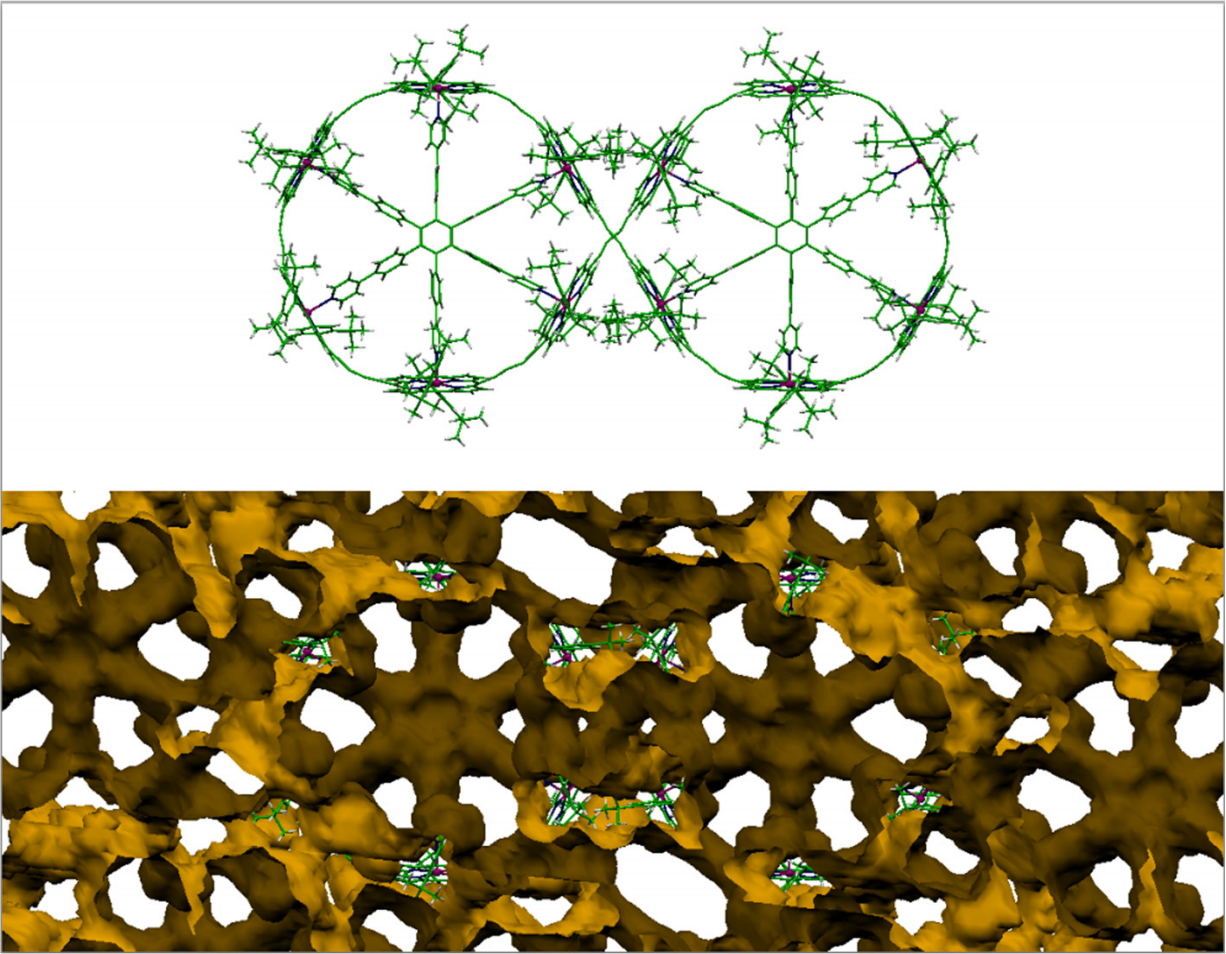
The structure of a double nano-wheel is shown (top) with the void space (below). The structure is 62 % solvent (calculated using PLATON [27])

A commonly used solution in the case of poorly resolved solvent is the BYPASS algorithm [26] as implemented in SQUEEZE within PLATON [27]. SQUEEZE uses the Fourier transform of the residual electron density in the void region to calculate a contribution to the structure factors. Historically crystallographic programs have implemented this as a correction to the magnitude of *Y*_*o*_ rather than adding magnitude and phase information to the calculated scattering. In CRYSTALS the latter approach has always been used and SQUEEZE output is integrated so that the complex structure factor of the solvent contribution is added to the *calculated* structure factors as shown in Equation 2. This approach is now also available in the latest release of SHELXL [28] *via* an additional *.fab* file output by SQUEEZE [29]. This approach is extremely powerful when dealing with complex materials as it enables the scientist to model the structure, then apply SQUEEZE which will lead to improvements in the atomic model. These improvements in turn affect subsequent estimates of $F_{c_{\text {solvent}}}$ by SQUEEZE, so iteratively refining the atomic model and running SQUEEZE will lead to a gradual improvements in the structure. Large molecule crystal structures can consist of as much as 60-80 % solvent by volume, far more than SQUEEZE was originally designed to deal with. Nevertheless, the approach works remarkably well. Without SQUEEZE, challenging structures like the porphyrin nano-wheels worked on by Anderson and co-workers [30, 31] would not have been soluble (Fig. 9).

### The developer Response to Larger Structures

One of the key problems associated with the refinement of large molecules is the amount of time it takes: in addition to the time spent building the model, dealing with restraints, checking the validity of the parameters *etc.*, there is the time spent by the computer actually processing the data. It may initially appear that computer processing is not the major bottleneck compared to the scientist actually modelling and refining a complex structure. However, while these computations of seconds or minutes are running the crystallographer is not free to do another task. It has been reported that users of software will lose attention and want to perform other tasks if forced to wait for more than ten seconds [32]. Studies consistently show that switching focus in this manner significantly impacts performance [33, 34] (regardless of a subject’s gender or degree of control over switching tasks [35]) and that the ability to switch tasks efficiently is actually negatively correlated with the subject’s own belief about their ability to multitask [36].

Improving the speed of the calculation during cycles of least-squares refinement therefore helps to improve the user experience with bigger structures, reducing frustration and time penalties associated with frequent task switching.

**Fig. 10:**
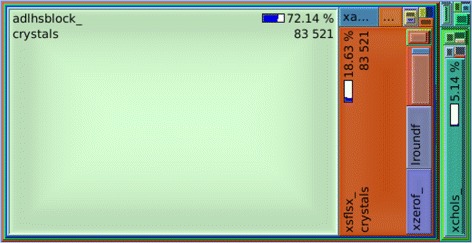
Graph displaying the number of instructions fetched by subroutines on the 14.4x version of CRYSTALS. The area is proportional to the number of instructions fetched. Results are also similar for data read cache misses. Refinement was carried out using 253 parameters and 4,913 reflections

The time taken for calculation in structure refinement is almost exclusively spent during the least-squares minimisation. There are three major computing parts during the minimisation (Fig. 10):

- calculation of the derivatives (xsflsx subroutine)
- formation of the normal matrix **AA**^*t*^ (adlhsblock subroutine)
- inversion of the normal matrix (xchols subroutine)

In versions of CRYSTALS 14.4x and earlier, the calculation of the derivatives (19 %) is relatively modest, the formation of the normal matrix (72 %) is the most time consuming part and the inversion (5 %) is negligible. However, these results were obtained on a very small structure of 253 parameters. Using a larger structure with 5,173 parameters, the inversion takes 17 % of the time while the calculation of the derivative is negligible at 1.5 %. The formation of the normal matrix remains the most consuming part at 78 %.^2^ The matrix inversion does not scale very well with the number of parameters (Fig. 13) hence the change in proportion. The time of the calculation depends simulatenously on the number of reflections and the number of parameters. The complexity increases linearly with the reflections, but with the parameters there is a much steeper increase. A small increase in the number of parameters results in a large increase in the calculation time, mainly due to the Cholesky inversion [37]. It should be noted that in general the number of parameters is proportional to the number of reflections, so that as larger molecules are studied both will tend to increase. We therefore chose to look for ways to improve the speed of two key parts of the algorithm: the formation of the normal matrix; and the matrix inversion.

#### Derivatives and normal matrix formation

Reflections are retrieved from a file one by one and the structure factor and its partial derivatives with respect to all parameters being refined are calculated. The derivatives, representing one row of the design matrix **A**, are used to update the normal matrix **N**. Updating the normal matrix reflection by reflection is a historical artefact, dating from an era when memory was a scarce resource. Nowadays, this approach is not optimal as it involves a large number of small i/o and memory transfers. Matrix multiplication is heavily bounded by memory bandwidth and less dependent on pure computing power [38]. The main difficulty is to feed data quickly enough to the computing units.

To address this in the latest version of CRYSTALS, reflections are loaded in batches. The derivatives are still calculated sequentially, but they are stored in a temporary partial design matrix block. The accumulation is done once the batch has been processed. This approach has the advantage that it groups similar operations together (i/o, computation of derivatives, matrix multiplications...), while the use of batches instead of the whole dataset maintains the flexibility to work with large sets of data that potentially would not fit in memory. In addition, the matrix multiplication to form the normal matrix is expensive enough to justify a call to an external dynamic library.

Further speed gains are made by optimising the calculation of the normal matrix (**AA**^*t*^), a special case of a general matrix multiplication where only half of the values need to be computed. In this case, much lower level modifications involving techniques such as blocking, loop unrolling, cache optimisation, vectorisation, *etc* are required. Optimisation is challenging as it decreases the readability and flexibility of the source code and the complexity of modern microprocessors makes it difficult for the developers to obtain and maintain skills in these areas. When writing their own software crystallographers can optimise their code to a certain extent, but it is very unlikely that they would match results of high performance libraries given the complexity of modern CPUs and the time they can afford for programming. For example, the simple task of multiplying two matrices efficiently requires a very complicated algorithm [39, 40] which has been refined over many years.

**Fig. 11:**
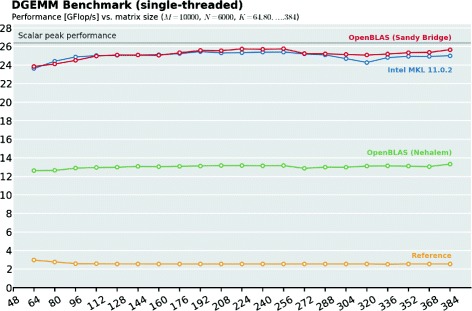
DGEMM subroutine’s performance on Intel Core i5-2500K Windows 7 SP1 64-bit. DGEMM is used internally in DSYRK. Reference is the reference BLAS implementation and the level that non-specialist usually achieve. (Credits: OpenBLAS wiki web page)

The gains are not marginal: high performance libraries can outperform any manual optimisation by several orders of magnitude (Fig. 11) and therefore it is pragmatic to make use of them. For this task CRYSTALS is using the subroutine DSYRK from the Level-3 BLAS (Basic Linear Algebra Subprogram) library. Several implementations exist, from microprocessor manufacturers or universities.

**Fig. 12:**
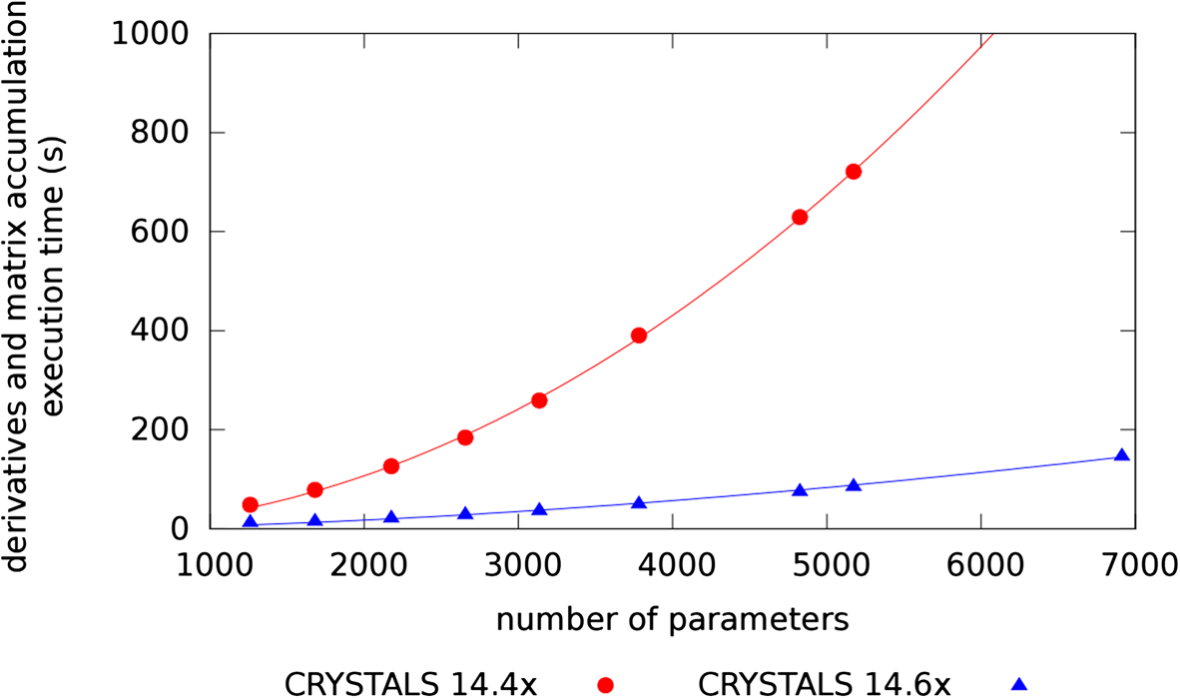
Time spent during the calculation of the derivatives and the normal matrix formation as a function of parameters

Figure 12 compares the performance before and after modification as a function of the parameters. The accumulation of equations of restraint into the normal matrix should also be optimised, however, because the design matrix rows are so sparse (a distance restraint, for example, only involves up to six parameters), the current implementation accumulating the restraints one by one is very efficient and BLAS routines are not used.

#### Matrix inversion

The second bottleneck spotted in CRYSTALS is the matrix inversion for solving the linearised problem. This operation is standard in the LAPACK [41], an extension of the Level-3 BLAS library. Several methods are available from Cholesky decomposition, **LDL**^*t*^ factorisation and eigen value decomposition. The home made Cholesky inversion used in CRYSTALS is replaced with an **LDL**^*t*^ factorisation via the calls to the subroutine SSYTRF and SSYTRI. SSYTRF uses the Bunch-Kaufman diagonal pivoting method for the factorisation. The new method is not only faster, but also scales much better with the number of parameters. Both the MKL and OpenBlas libraries are multi-threaded and will exploit multiple CPU cores when present.

The gains in performance gave us the opportunity to implement two features which will improve precision while the time penalties involved are masked by the overall gains.

Firstly, small numerical errors can occur when using single precision in the derivative and structure factor calculations. Therefore the accumulation is done in double precision^3^.

Secondly, the normal matrix can be pre-conditioned before inversion. Pre-conditioning improves the accuracy of the inversion by reducing the loss of precision during floating point operations due to truncation or round-off errors [42]. Pre-conditioning is applied as a pre-multiplication and post-multiplication by the diagonal matrix **C** on the matrix to invert, **N**.

**C** is chosen so that the new resulting matrix *N*^′^ has all its diagonal elements equal to one. The inverse matrix **N**^−1^ is recovered by using the matrix **C** again:

(3) $$ \mathbf{N^{-1}} = \mathbf{CN'^{-1}C}  $$

**Fig. 13:**
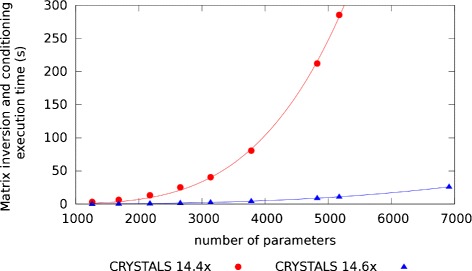
Time spent during the inversion as a function of parameters

Overall, the results obtained from all of these modifications are extremely beneficial, especially the inversion which now scales much better than the old method with an increasing number of parameters: Using 1,261 parameters, the new method is 18 times faster. With 5,173 parameters, the inversion is 26 times faster (Fig. 13). The previous implementation of matrix inversion in CRYSTALS attempted to eliminate singularities from ill-conditioned problems, but at some computational cost and poor scalability with problem size. The current routine will fail with a warning if a singular matrix is encountered and the user can instead attempt inversion using eigenvalue filtering to temporarily ignore the problem.

#### Global optimisation and comparisons

An attempt to parallelise the calculation of the derivatives and the normal matrix is in progress, but due to historical constraints in the source code, for now, only a minor part surrounding the normal matrix accumulation has been improved. Parallelisation of the matrix inversion is automatically dealt with within the LAPACK library. Overall, CRYSTALS benefits from having a small number of additional CPU cores available, but scalability is far from ideal.

**Fig. 14:**
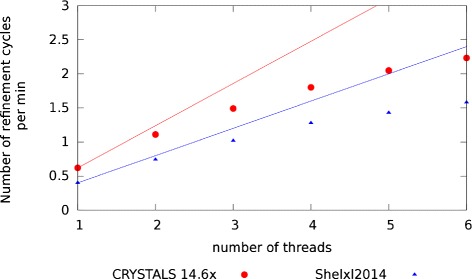
Multi-cores performance and comparison with SHELXL2014. Structure used was using 5,173 parameters and 49,666 reflections. The straight line is the theoritical speed-up based on the single core performance

A comparison has been run with the latest version of SHELXL [28] available (29th of July 2014). The data used for the comparison contains 49,666 reflections and 5,173 parameters. The result is shown in Fig. 14. Historically CRYSTALS was noticeably slow when refining very large structures, but this new version brings it up to the same performance as similar software packages. Using a single core, CRYSTALS is 50 % faster that SHELXL in this particular test case. Although SHELXL has still the advantage of scaling better when using multiple cores, the performance gap disappears when the size of the least-squares problem decreases.

The old Fortran code in crystals is slowly being converted to modern Fortran to ensure a smooth transition to the future. This will enable further improvements and new optimisations in the future.

## Conclusions

We have shown that the size of structures being published is increasing, and that this is directly correlated with the occurrence of disorder. Disorder leads to more manual effort constructing suitable structural models while the larger size of the optimisation problem leads to ever slower refinements. The developments within CRYSTALS, described above, are helping to keep pace with these changes. There are still some areas in CRYSTALS where some improvements are needed. The scalability using multi-threads is probably going to give a significant improvement in the near future. Reorganisation of the code to allow a better vectorisation should also give good results on modern CPUs. This is also where the current micro-processors manufacturers improve their CPUs.

## Methods

Benchmarks have been realised on a 8 cores Intel®; Xeon®; CPU E5-2665 with the Hyper-Threading Technology and Turbo Boost Technology deactivated. This CPU includes the latest 256 bits vector instructions AVX which have been used in crystals during the benchmarks (SHELXL does not seem to use these instructions). The operating system was Centos Linux 6.5 in 64 bits. CRYSTALS is open source and free to use, as such the source code can be downloaded from the website [43]. The new implementation of the software described in this publication is present in revision 5473 and earlier under subversion. CRYSTALS was compiled using the Gnu compiler collection (gcc) 4.9 and the library OpenBlas 0.2.11 [44]. At present, the Windows build of CRYSTALS is using Intel®; Math Kernel Library (Intel®; MKL) which offers similar performance to OpenBlas. SHELXL2014 64 bits was used as provided by the author’s website. The dataset used for the refinements is from Kondratuk D. V. *et al.* [30] with 49,666 reflections. The number of parameters refined varies between 1,261 to 6,913 depending on the test.

Profiling of the code used Valgrind, an instrumentation framework for building dynamic analysis tools [45] and kcachegrind as a visualisation software. Due to a huge overhead, only small systems up to a few hundreds parameters can be used. For bigger molecules, *perf*, a Linux profiling with hardware performance counters is used with virtually no impact on the execution time.

Time measurements made on CRYSTALS were made using timers in the source code around the area of interest. For SHELXL, the refinement cycle duration was taken as the difference between two timesamps printed during the refinement.

## Endnotes

^1^ An assembly, as defined by the International Union of Crystallography’s CIF dictionary is “a cluster of atoms that show long-range positional disorder but are locally ordered. Within each such cluster of atoms [a group] is used to identify the sites that are simultaneously occupied”.

^2^ These values were not obtained with Valgrind but using *perf* which has very little overhead performance penalty.

^3^ Multiplication and addition have the same cost either for single precision or double precision. However, vector units can hold half the number of double precision numbers hence the performance penalty.
